# miR-485 inhibits histone deacetylase HDAC5, HIF1α and PFKFB3 expression to alleviate epilepsy in cellular and rodent models

**DOI:** 10.18632/aging.203058

**Published:** 2021-05-21

**Authors:** Wei Pan, Xingyu Song, Qibo Hu, Yunfeng Zhang

**Affiliations:** 1 Department of Pediatrics, The Second Hospital of Jilin University, Changchun 130041, P.R. China

**Keywords:** epilepsy, apoptosis, miR-485, HDAC5, HIF1α

## Abstract

We investigated the role of microRNA (miR)-485 and its downstream signaling molecules on mediating epilepsy in cellular and rat models. We established a cellular epilepsy model by exposing hippocampal neurons to magnesium and a rat model by treating ICR mice with lithium chloride (127 mg/kg) and pilocarpine (30 mg/kg). We confirmed that miR-485 could bind and inhibit histone deacetylase 5 (HDAC5) and then measured expression of miR-485 and in mice and cells. Cells were transfected with overexpression or knockdown of miR-485, HDAC5, hypoxia-inducible factor-1alpha (HIF1α), or 6-phosphofructo-2-kinase/fructose-2,6-biphosphatase 3 enzyme (PFKFB3) to verify their roles in apoptosis, oxidative stress, and inflammation in epileptic hippocampal neurons. Binding relationship between miR-485, HDAC5, HIF1α, and PFKFB3 was verified. Oxidative stress and inflammation marker levels in epilepsy model mice were assessed. miR-485 was downregulated and HDAC5 was upregulated in cell and animal model of epilepsy. Seizure, neuronal apoptosis, oxidative stress (increased SOD and GSH-Px expression and decreased MDA and 8-OHdG expression) and inflammation (reduced IL-1β, TNF-α, and IL-6 expression) were reduced by miR-485 in epileptic cells. HIF1α and PFKFB3 expression was reduced by HDAC5 knockdown in cells, which was recapitulated *in vivo*. Thus, miR-485 alleviates neuronal damage and epilepsy by inhibiting HDAC5, HIF1α, and PFKFB3.

## INTRODUCTION

Epilepsy is defined clinically by at least two unprovoked seizures in more than 24 hours according to the International League Against Epilepsy [1]. Today, epilepsy affects 50 to 70 million people worldwide [1, 2], with an estimated prevalence of 2.2 to 41.0 per 1000 people worldwide [3]. Major causes of epilepsy include, but are not limited to, birth trauma, cerebrovascular disease, infections of the central nervous system, and head injuries [4]. The treatment of epilepsy remains expensive [5] and unsatisfactory, despite the availability of more than 20 anti-epileptic medications [2], and surgical procedures. The incidence in China is similar to that in Europe and the USA [4], and has increased from two to seven per 1000 people [6]. This increasing prevalence may reflect improved diagnosis in China, but nonetheless alerts to the need for a better understanding of the mechanisms leading to epilepsy.

Non-coding microRNAs (miRs) are well-known to be involved in epilepsy. For example, miR-134 has anti-seizure and disease-modifying abilities in epilepsy [7]. In fact, miR-124, miR-128, and miR-199 are other good candidates as diagnostic or prognostic biomarkers of epilepsy [8, 9]. A recent study revealed decreased cerebral expression of miR-485 in an epilepsy model [10], but the involvement of miR-485 in human epilepsy remains to be established Therefore, we evaluated the role of miR-485 in epilepsy in cell and animal models.

Histone deacetylase 5 (HDAC5) broadly influences transcriptional regulation because histone deacetylation alters chromosomal structure to regulate the access of transcription factors to DNA. This presents a mechanism by which miRNAs can alter gene expression through effects on HDAC5. For example, miR-124 and miR-9 mediate downregulation of HDAC5, which ultimately promotes neurite development [11]. Following upon that precedent, we proposed in this study to investigate a possible relationship between miR-485 and the expression of HDAC5, which we predict to be a downstream signaling molecule of miR-485-related epilepsy.

Mounting evidence from numerous pre-clinical and clinical studies suggests that long-lasting inflammatory processes involving cytokines and other proinflammatory mediators in brain promote neuronal death, increase neuronal excitability, and lower the threshold for seizure [12]. Oxidative stress rises from any imbalance between production of reactive oxygen species or free radicals in living cells and tissues, and the capacity for their detoxification. Oxidative stress is a factor in the propagation of epilepsy [13], which can establish a neurotoxic feedback look where seizures increase oxidative stress and neuroinflammation, this exacerbating the underlying seizure disorder [14], ultimately contributing to cell necrosis and apoptosis [15].

Hypoxia-inducible factor-1alpha (HIF1α) is a pro-inflammatory downstream mediator of HDAC5 [16, 17], which can promote epilepsy [18]. Moreover, 6-phosphofructo-2-kinase/fructose-2, 6-biphosphatase 3 enzyme (PFKFB3) is among the downstream targets of HIF1α [19]. Given the involvement of PFKFB3 is involved in neuronal excitotoxicity, which is an important mechanism for epilepsy [20, 21], we set about to investigate the interactions among miR-485, with HDAC5, HIF1α, and PFKFB3 in cellular and animal models of epilepsy.

## RESULTS

### miR-485 overexpression alleviates epilepsy in mice

**Figure 1:**
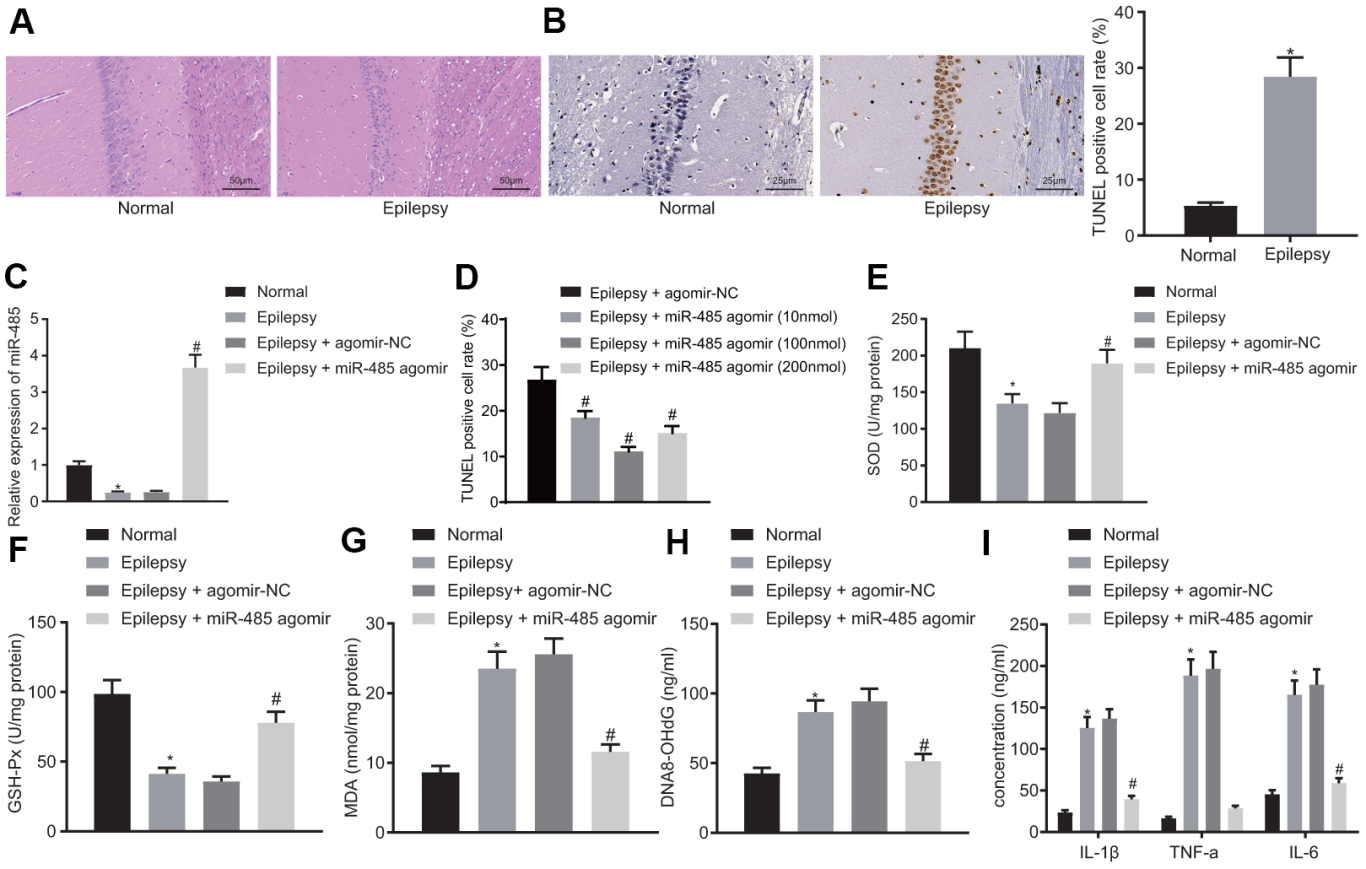
**miR-485 overexpression attenuates epilepsy.** (**A**) Representative HE micrographs showing histopathological changes in the hippocampus (200 ×). (**B**) Cell apoptosis in the hippocampus determined by TUNEL assay (400 ×). (**C**) miR-485 expression in mouse brain tissues determined by RT-qPCR. (**D**) Cell apoptosis in the hippocampus determined by TUNEL assay (400 ×). (**E**) SOD levels. (**F**) GSH-Px levels. (**G**). MDA levels. (**H**) DNA 8-OHdG levels. (**I**) IL-1β, TNF-α, IL-6 levels. * *p* < 0.05 vs. control mice; # *p* < 0.05 vs. epileptic mice treated with agomir-NC; n = 10. Data were expressed as mean ± standard deviation. Data between two groups were compared independent sample t-test. Data among multiple groups were compared by one-way analysis of variance and post-hoc Tukey’s test.

Normal mice had no seizures (level 0). Epilepsy model mice had seizures in classes III-V, with the preponderance in the classes IV-V (Table 1). HE staining in normal mice showed abundant pyramidal cells in the hippocampus, but there was significant damage to these neurons in epileptic mice, with neuronal loss, and disrupted organization and cell structure of the remaining neurons (Figure 1A). In hippocampus from epileptic mice, TUNEL-positive (apoptotic) cells were significantly increased compared to normal mice (Figure 1B), thus histologically confirming the epilepsy model.

**Table 1:** Racine seizure classification in all groups of mice.

**Groups**	**Cases (n)**	**0 grade**	**I grade**	**II grade**	**III grade**	**IV grade**	**V grade**
Normal	10	10	0	0	0	0	0
Epilepsy	10	0	0	0	1	2	7
Epilepsy + agomir-NC	10	0	0	0	2	2	6
Epilepsy + miR-485 agomir	10	6	3	1	0	0	0

miR-485 expression was decreased in brain tissues from epilepsy model mice compared to normal brain (Figure 1C). miR-485 agomir, as expected, increased miR-485 expression in epileptic mice (Figure 1C), and decreased the seizure grade to 0-II (Table 1). miR-485 agomir also reduced apoptosis (Figure 1D) in a dose-dependent manner. Based on initial results, we selected a dose of 100 nmol miR-485 agomir for the main part of the study. In the background of epilepsy research, SOD, GSH-Px, and MDA are often used as markers of oxidative stress responses [22, 23]. Present results indicated decreased levels of SOD (Figure 1E) and GSH-Px (Figure 1F) in epilepsy mice, whereas there were increased concentrations of MDA (Figure 1G) and hippocampal DNA 8-OHdG (Figure 1H) compared to levels in normal mice. These markers were normalized by treatment with miR-485 agomir. Expression of proinflammatory cytokines IL-1β, TNF-α, and IL-6 was increased in epileptic mice compared to control mice (Figure 1I). However, miR-485 agomir rescued these effects, thus consistently indicating that miR-485 overexpression alleviated manifestations of epilepsy in the present murine model.

### miR-485 inhibits apoptosis, oxidative stress, and inflammation by targeting HDAC5 in epileptic hippocampal neurons

**Figure 2:**
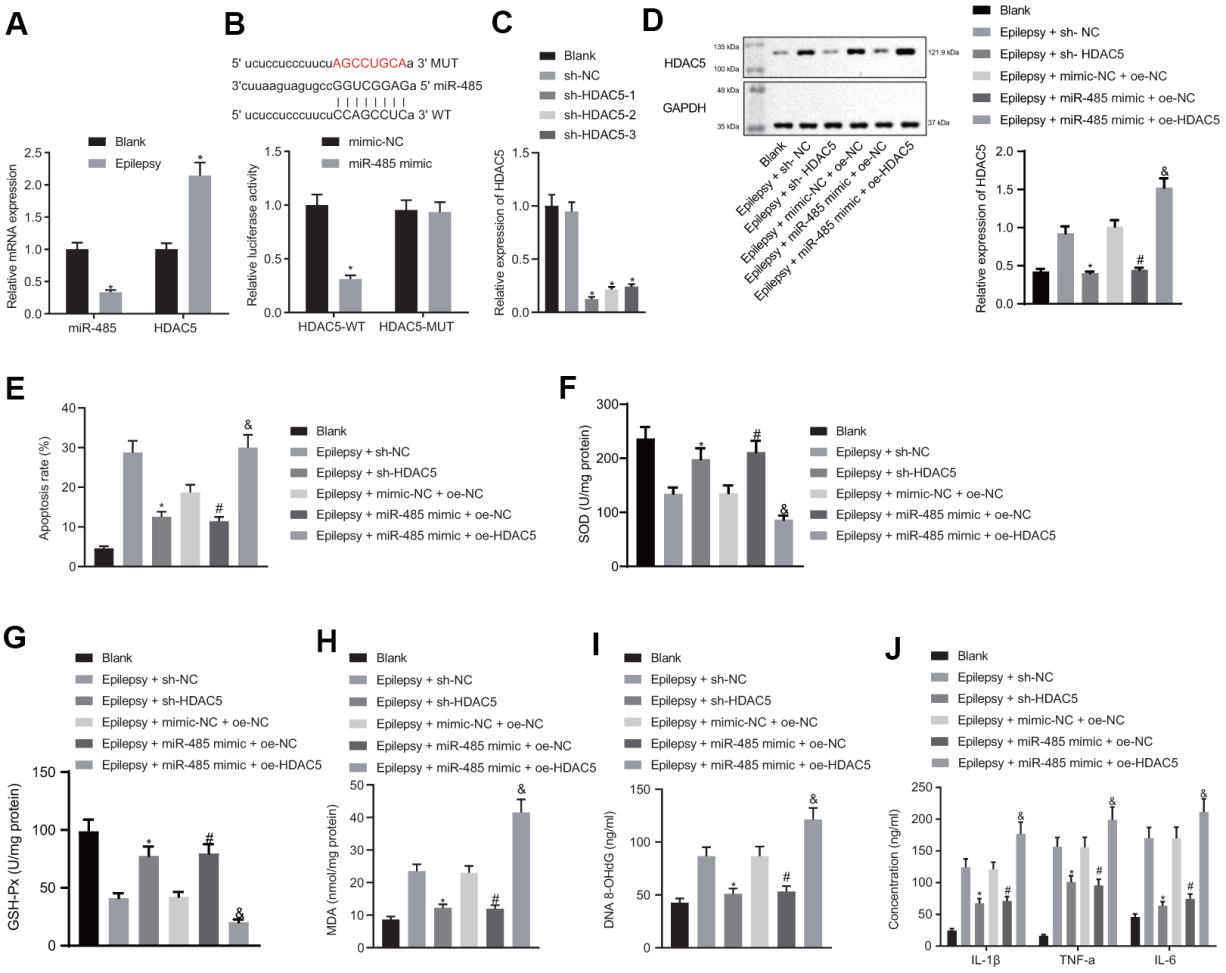
**miR-485 repressed apoptosis, oxidative stress and inflammation by targeting HDAC5 in epilepsy model hippocampal neurons.** (**A**) miR-485 and HDAC5 mRNA expression. (**B**) Binding relationship between miR-485 and HDAC5 determined by dual luciferase reporter gene assay. (**C**) Efficiency of HDAC5 knockdown. (**D**) HDAC5 protein expressions determined by western blotting. (**E**) Cell apoptosis determined by flow cytometry. (**F**) SOD levels. (**G**) GSH-Px levels. (**H**) MDA levels. (**I**) DNA 8-OHdG levels. (**J**) IL-1β, TNF-α, IL-6 levels. Data are expressed as mean ± standard deviation. * *p* < 0.05 vs. blank cells, cells treated with mimic-NC, or epilepsy cells treated with sh-NC; # *p* < 0.05 vs. epilepsy cells treated with mimic-NC + oe-NC; ^&^ *p* < 0.05 vs. epilepsy cells treated with miR-485 mimic + oe-NC. Data between two groups were compared independent sample t-test. Data among multiple groups were compared by one-way analysis of variance and post-hoc Tukey’s test. Experiments were repeated three times.

miR-485 expression was decreased in the hippocampal neuron model of epilepsy (Figure 2A). microRNA.org and Starbase predicted that miR-485 targeted HDAC5 both in human and mouse, and we found increased HDAC5 expression in the hippocampal neuron model of epilepsy (Figure 2A). Dual luciferase reporter gene assay confirmed the predicted binding relationship: miR-485 mimic reduced the wild-type luciferase activity in the HDAC5 3'UTR region, but had no effect on the mutant type (Figure 2B). sh-HDAC5-1, sh-HDAC5-2, and sh-HDAC5-3 all reduced HDAC5 expression, with the greatest effect in sh-HDAC5-1, which was consequently selected for subsequent experiments (Figure 2C). HDAC5 silencing decreased HDAC5 expression in the hippocampal neuron model of epilepsy, as did treatment with miR-485 mimic, an effect which was reversed by HADC5 overexpression (Figure 2D). HDAC5 silencing or miR-485 mimic reduced apoptosis in epilepsy model neurons, while HDAC5 overexpression reversed the effect of miR-485 mimic (Figure 2E). HDAC5 silencing or miR-485 mimic increased SOD (Figure 2F) and GSH-Px (Figure 2G) expression, while decreasing the concentrations of MDA (Figure 2H) and DNA 8-OHdG (Figure 2I) in epilepsy model cells. These effects of miR-485 mimic were inhibited by HDAC5 overexpression. HDAC5 silencing or miR-485 mimic reduced the formation of IL-1β, TNF-α, and IL-6 by epilepsy model neurons, and this effect was rescued by HDAC5 overexpression (Figure 2J). Taken together, these results indicate that miR-485 inhibited apoptosis, oxidative stress, and inflammation by targeting and inhibiting HDAC5 in epilepsy model neurons.

### HDAC5 promotes epilepsy through upregulating HIF1α and PFKFB3 in hippocampal neurons

**Figure 3:**
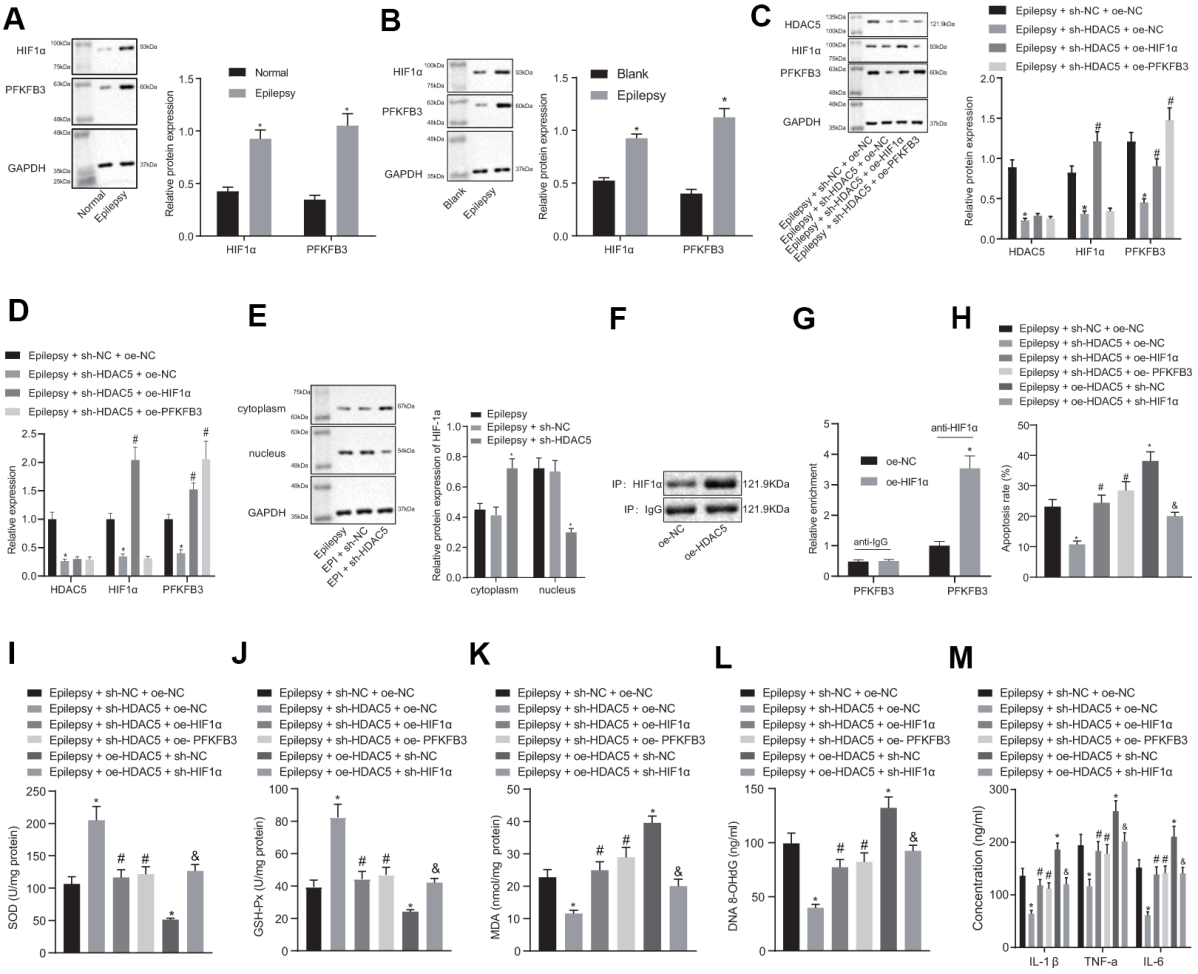
**HDAC5 results in promotion of epilepsy via HIF1α and PFKFB3 upregulation in epilepsy hippocampal neurons.** (**A**) HIF1α and PFKFB3 protein expression in mice (n = 10). (**B**) HIF1α and PFKFB3 protein expression in cells. (**C**) Protein expression of HDAC5, HIF1α, and PFKFB3 in cells. (**D**) mRNA expression of HDAC5, HIF1α, and PFKFB3 in cells. (**E**) HIF1α protein expression in the nucleus and cytoplasm. (**F**) IP detected the binding of HDAC5 to HIF1α. (**G**) CHIP detected the enrichment of HIF1α in PFKFB3 promoter. (**H**) Cell apoptosis determined by flow cytometry. (**I**) SOD levels. (**J**) GSH-Px levels. (**K**) MDA levels. (**L**) DNA 8-OHdG levels. (**M**) IL-1β, TNF-α, and IL-6 levels. * *p* < 0.05 vs. control cells, blank control cells, epilepsy cells treated with sh-NC, or epilepsy cells treated with sh-NC + oe-NC; # *p* < 0.05 vs. epilepsy cells treated with sh-HDAC5 + oe-NC. Data were expressed as mean ± standard deviation. Data between two groups were compared independent sample t-test. Data among multiple groups were compared by one-way analysis of variance and post-hoc Tukey’s test. Experiments were repeated three times.

Expression of HIF1α and PFKFB3 expression was elevated in epileptic mice and cell models (Figure 3A, 3B), but HDAC5 silencing reduced HIF1α and PFKFB3 expression in epileptic cells. The HIF1α overexpression did not affect HDAC5 expression, but increased HIF1α and PFKFB3 levels in the presence of sh-HDAC5. Moreover, the PFKFB3 overexpression increased PFKFB3 expression only in the presence of sh-HDAC5 (Figure 3C, 3D). HDAC5 silencing resulted in a reduction of HIF1α nucleation (Supplementary Figure 1) and HIF1α expression (Figure 3E) in the nucleus. IP detected the binding of HDAC5 to HIF1α, and the results showed that oe-HDAC5 combined more HIF1α than oe-NC (Figure 3F). CHIP detected the enrichment of HIF1α in PFKFB3 promoter. The results showed that compared with oe-NC, the enrichment of oe-HIF1α in PFKFB3 promoter region was significantly increased (Figure 3G).

HDAC5 silencing reduced apoptosis in epilepsy model cells, which was reversed by HIF1α or PFKFB3 overexpression (Figure 3H). HDAC5 silencing enhanced SOD (Figure 3I) and GSH-Px (Figure 3J) expression, but decreased MDA (Figure 3K), DNA 8-OHdG (Figure 3L), and IL-1β, TNF-α and IL-6 levels (Figure 3M) in epileptic cells. These effects of HDAC5 silencing were abrogated by HIF1α or PFKFB3 overexpression. In addition, overexpression of HDAC5 increased apoptosis, decreased SOD and GSH-Px contents, increased MDA and DNA 8-OHdG levels, and increased IL-1β, TNF-α, and IL-6 levels, which were reversed by further downregulation of HIF1α. These results consistently suggest that HDAC5 promoted apoptosis, oxidative stress, and inflammation via upregulation of HIF1α and PFKFB3.

### miR-485 inhibits apoptosis, oxidative stress, and inflammation through HDAC5/HIF1α/PFKFB3 downregulation in epilepsy model cells

**Figure 4:**
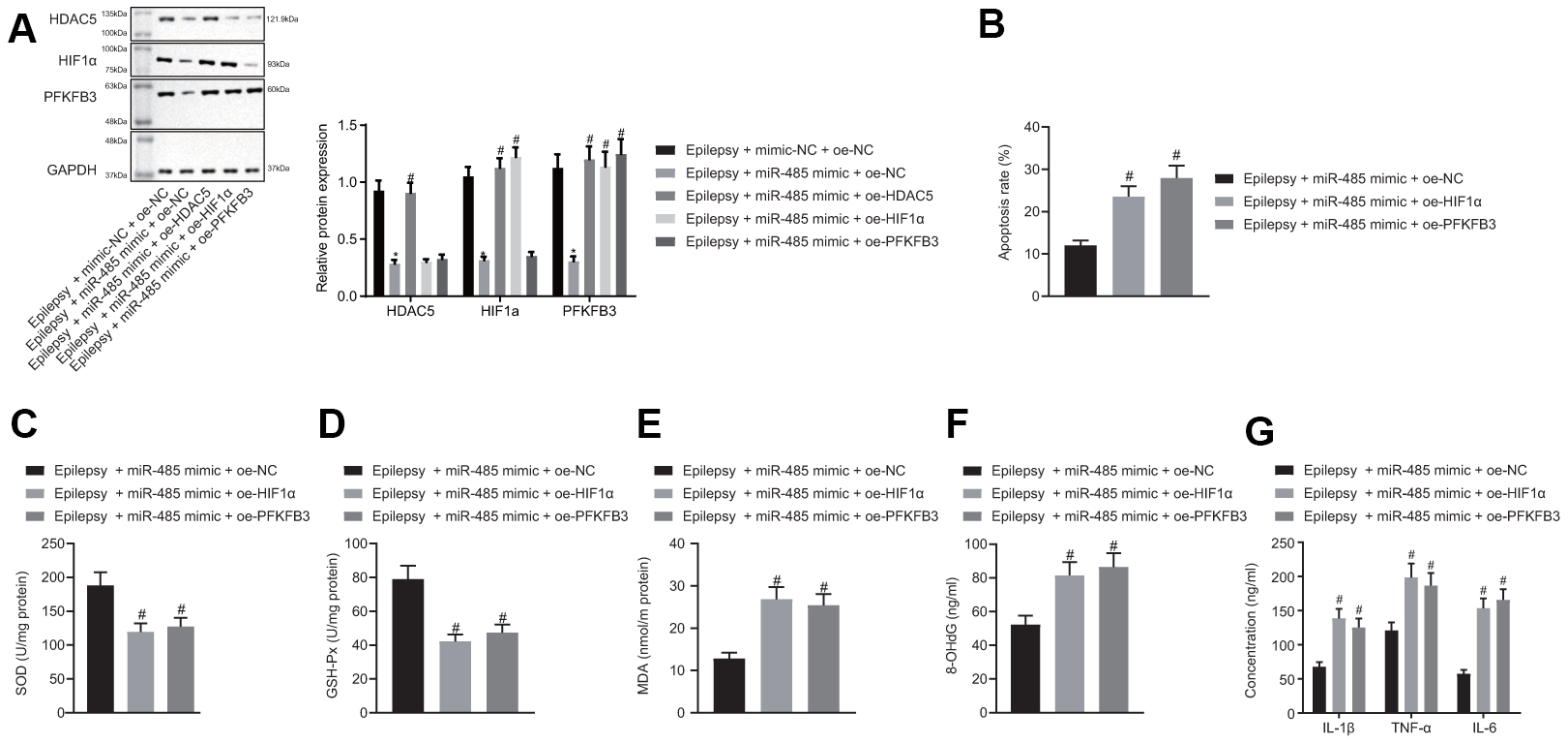
**miR-485 suppresses apoptosis, oxidative stress, and inflammation through HDAC5/HIF1α/PFKFB3 upregulation in epilepsy hippocampal neurons.** (**A**) Protein expression of HDAC5, HIF1α, and PFKFB3. (**B**) Cell apoptosis determined by flow cytometry. (**C**) SOD levels. (**D**) GSH-Px levels. (**E**) MDA levels. (**F**) DNA 8-OHdG levels. (**G**) IL-1β, TNF-α, and IL-6 levels. * *p* < 0.05 vs. epilepsy cells treated with mimic-NC + oe-NC; # *p* < 0.05 vs. epilepsy cells treated with miR-485 mimic + oe-NC. Data were expressed as mean ± standard deviation. Data between two groups were compared independent sample t-test. Data among multiple groups were compared by one-way analysis of variance and post-hoc Tukey’s test. Experiments were repeated three times.

miR-485 mimic reduced HDAC5, HIF1α, and PFKFB3 expressions in epilepsy model cells. The forced HDAC5 overexpression increased HDAC5, HIF1α, and PFKFB3 expression in the presence of miR-485 mimic in epilepsy model cells, but increased HIF1α and PFKFB3 expressions only in the presence of miR-485 mimic. Furthermore, PFKFB3 overexpression increased PFKFB3 expression only in the presence of miR-485 mimic (Figure 4A), whereas miR-485 mimic, HIF1α, or PFKFB3 overexpression the increased apoptosis (Figure 4B). HIF1α or PFKFB3 overexpression reduced SOD (Figure 4C) and GSH-Px (Figure 4D) concentration, but increased MDA (Figure 4E), DNA 8-OHdG (Figure 4F), and IL-1β, TNF-α, and IL-6 levels (Figure 4G) in the presence of miR-485 mimic. Overall, miR-485 inhibited apoptosis, oxidative stress, and inflammation in epilepsy model cells through the HDAC5/HIF1α/PFKFB3 axis.

### miR-485 inhibits epilepsy through HDAC5/HIF1α/PFKFB3 axis *in vivo*

**Figure 5:**
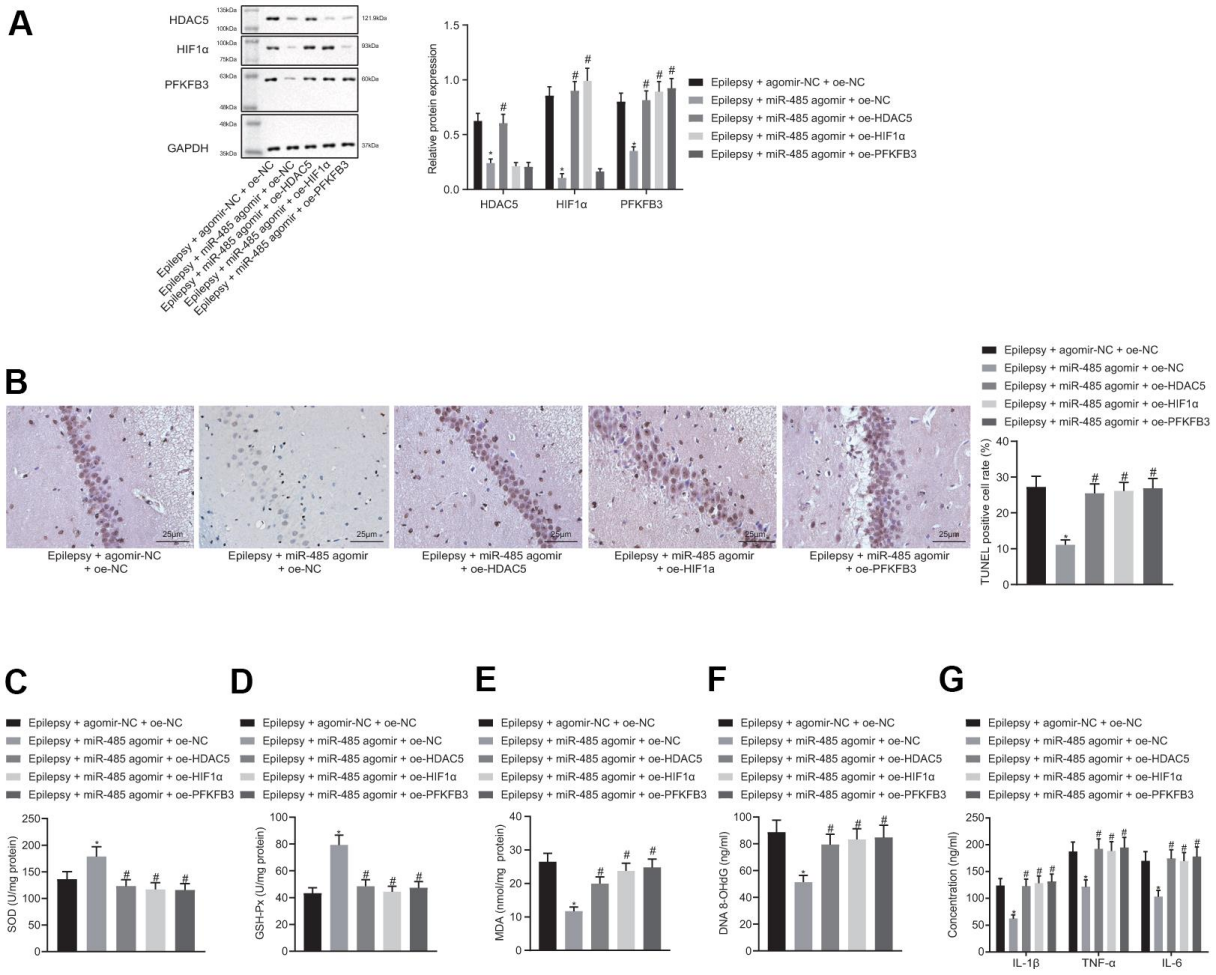
**miR-485 inhibits epilepsy through HDAC5/HIF1α/PFKFB3 axis *in vivo*.** (**A**) Protein expression of HDAC5, HIF1α, and PFKFB3 in the brain tissues. (**B**) Cell apoptosis determined by TUNEL assay (400 ×). (**C**) SOD levels. (**D**) GSH-Px levels. (**E**) MDA levels. (**F**) DNA 8-OHdG levels; (**G**) IL-1β, TNF-α, IL-6 levels. * *p* < 0.05 vs. epileptic mice treated with agomir-NC + oe-NC; # *p* < 0.05 vs. epileptic mice treated with miR-485 agomir + oe-NC; n = 10. Data were expressed as mean ± standard deviation. Data among multiple groups were compared by one-way analysis of variance and post-hoc Tukey’s test.

miR-485 agomir decreased HDAC5, HIF1α and PFKFB3 expression in brain of epileptic mice. In the presence of miR-485 agomir, the HDAC5 overexpression increased HDAC5, HIF1α, and PFKFB3 expression only in epileptic mice. (Figure 5A). The results of immunohistochemistry were consistent with that of Western blot (Supplementary Figure 2). miR-485 agomir decreased seizure grades from IV-V down to I-II, which was reversed by HDAC5, HIF1α, or PFKFB3 overexpression (Table 2). miR-485 agomir reduced apoptosis (Figure 5B), but these effects were reversed by HDAC5, HIF1α, or PFKFB3 overexpression. Moreover, miR-485 agomir increased SOD (Figure 5C) and GSH-Px (Figure 5D) contents, but decreased MDA (Figure 5E), DNA 8-OHdG (Figure 5F), and IL-1β, TNF-α and IL-6 levels (Figure 5G) in epileptic mice, all of which was abolished by HDAC5, HIF1α, or PFKFB3 overexpression. Taken together, these results show that miR-485 inhibited epilepsy through the HDAC5/HIF1α/PFKFB3 axis *in vivo*.

**Table 2:** Racine seizure classification in all groups of mice.

**Groups**	**Cases (n)**	**0 grade**	**I grade**	**II grade**	**III grade**	**IV grade**	**V grade**
Epilepsy + agomir-NC + oe-NC	10	0	0	0	1	1	8
Epilepsy + miR-485 agomir + oe-NC	10	0	6	4	0	0	0
Epilepsy + miR-485 agomir + oe-HDAC5	10	0	0	0	3	2	5
Epilepsy + miR-485 agomir + oe-HIF1α	10	0	0	0	2	2	6
Epilepsy + miR-485 agomir + oe-PFKFB3	10	0	0	0	1	1	8

## DISCUSSION

**Figure 6:**
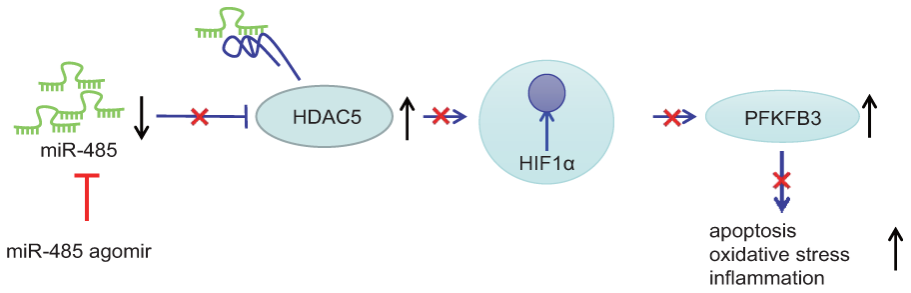
miR-485 alleviates epilepsy through inhibition of HDAC5, leading to downregulation of HIF1α and PFKFB3.

A key finding of this study is that expression of miR-485 expression was reduced in epilepsy model neurons and in brain of epileptic mice. Conversely, miR-485 overexpression reduced seizure, neuronal apoptosis, oxidative stress, and inflammation. We also confirmed the hypothesis that miR-485 bound to and inhibited HDAC5. Furthermore, HDAC5 silencing decreased the expression of HIF1α and PFKFB3. Finally, the anti-seizure, anti-apoptotic, anti-oxidative, and anti-inflammatory effects of miR-485 overexpression was inhibited by either HIF1α or PFKFB3 overexpression. These results held equally in the epilepsy neuron model *in vitro* and the animal model *in vivo*. Collectively, we find that miR-485 alleviates epilepsy by inhibiting HDAC5, HIF1α, and PFKFB3 expression, thus presenting therapeutic targets for the treatment of epilepsy (Figure 6). Present results concur with findings of a previous study revealing low expression of miR-485-5p in epilepsy [10]. Furthermore, other miRs such as miR-134, miR-181a, miR-124, miR-199a, and miR-128, have all exhibited low expression in epilepsy, thus presenting a broad spectrum of miRNAs as candidates for diagnostic biomarkers of epilepsy [8]. Moreover, we found that miR-485 alleviated seizures in the pilocarpine epilepsy model and reduced neuronal injury, drawing special attention to the potential of miR-485 as a novel therapeutic target for treating epilepsy.

We proceeded in our study to investigate the downstream signaling molecules involved in miR-485-related epilepsy, which showed that miR-485 bound to and inhibited HDAC5 expression. This seems to be the first such demonstration, but stands in line with a previous study showing regulation of HDAC5 by other miRs, whereby HDAC5 emerges as a cellular conductor of MEF2C and M6a activity, under the regulation of miR-124 and miR-9 in the control of neurite development [11]. HDAC5 acts as a transcriptional factor that affects cell function through its effects on histone deacetylation. In this study, we also showed that HDAC5 can have excitotoxic effects in neurons, as shown by our finding that HDAC5 knockdown decreased apoptosis, oxidative stress, and inflammation in neurons. The apoptotic effects of HDAC5 has been reported previously in a study showing that HDAC5 overexpression promotes proliferation but decreases caspase-dependent apoptosis in hepatocellular carcinoma cell lines [24]. Likewise, another previous study showed that the pro-inflammatory effect of HDAC5 may work through the activation of NF-KB [25].

Subsequently, we found that HDAC5 upregulated HIF1α in the nucleus of neurons. A prior study has also shown that HIF1α is a downstream mediator of HDAC5 [16, 17]. Importantly, we found that that HIF1α overexpression reversed the anti-seizure, anti-apoptotic, anti-oxidative stress, and anti-inflammation effects of miR-485. These results are supported by earlier findings that HIF1α regulates the Notch pathway to enhance neurogenesis in acute epilepsy, thus presenting another potential therapeutic target for epilepsy [18]. Seizure commonly causes hypoxia in neurons [26, 27], and hypoxia and HIF1α are well-known to cause increased oxidative stress and inflammation in the brain [28, 29]. Therefore, we provide strong evidence that HIF1α is a mediator in the pathway that promotes neuronal injury during epilepsy.

Previous works show that PFKFB3 is involved in neuronal excitotoxicity, which is an important mechanism for epilepsy [20, 21]. In this study, we found that HIF1α upregulated PFKFB3, which has also been shown previously in a study reporting the suppression of PFKFB3 through inhibiting of the HIF1α accumulation mediated by metformin [19]. Similar to the findings with HIF1α, PFKFB3 overexpression significantly reduced the anti-seizure effects and neuronal protective effects of miR-485. Previous studies have demonstrated a role of PFKFB3 on apoptosis, oxidative stress, and inflammation in various pathological conditions. Furthermore, PFKFB3 knockdown significantly inhibited TNF-α-induced endothelial inflammation [30]. Also, PFKFB3 is involved in alleviating sepsis-related acute lung injury via suppressing inflammation and apoptosis of alveolar epithelial cells [31]. Adding to this base, we now present strong evidence that PFKFB3 regulation is also involved in epilepsy.

In conclusion, miR-485 alleviates epilepsy through inhibition of HDAC5, leading to downregulation of HIF1α and PFKFB3. This study provides important new information concerning the pathophysiological mechanisms of epilepsy, thus drawing attention to a signaling pathway with potential as a new therapeutic target that merits further investigations. We note that our use of chemically-induced epilepsy cell and animal models present certain limitations. Although these models are widely used in research, the natural causes of epilepsy are diverse, including birth trauma, cerebrovascular disease, infections of the central nervous system and head injuries [4]. Therefore, we intend to test the generalizability of present findings in other epilepsy models.

## MATERIALS AND METHODS

### Ethics approval

The experiments involving animals were implemented in accordance with the principles embodied in the National Institutes of Health Guide for the Care and Use of Laboratory. Efforts were made to minimize animal numbers and discomfort.

### Mouse model of epilepsy

Male ICR mice (25–30 g, 6-week-old, n=90, Charles River, Beijing, China) was kept in animal facility under 12 h light/dark cycle at 22–25° C with free access to food and water. Stereotactic injections were performed on mice as described previously [32]. Animals were divided into nine groups (n=10/group): control, epilepsy, epilepsy + agomir-NC, epilepsy + miR-485 agomir, epilepsy + agomir-NC + oe-NC, epilepsy + miR-485 agomir + oe-NC, epilepsy + miR-485 agomir + oe-HDAC5, epilepsy + miR-485 agomir + oe-HIF1α, and epilepsy + miR-485 agomir + oe-PFKFB3. Mice were anesthetized with 2.5% sodium pentobarbital (10 mg/kg, i.p.) and secured on a brain stereotaxic instrument. The scalp was disinfected with 75% alcohol and an incision measuring 0.5 cm long made at the midline. After sterilizing the skull surface with 0.05% hydrogen peroxide, we made a 0.8 mm drill hole the stereotaxic position (-2.18, -2.6, -2.5) [32]. We made injections of the lentivirus vectors (0.5 μL/min) lasting six min into the hippocampus using a 2A micro syringe. The scalp was sutured, and mice were kept warm until returning to the cage. Lentiviral vector was provided by GeneChem (Shanghai, China). Two weeks after stereotactic injection of lentiviral vectors, control mice were treated with lithium chloride (127 mg/kg, i.p.) followed by saline vehicle 20 h later. Epileptic mice received lithium chloride (127 mg/kg, i.p.) followed 20 hours later by pilocarpine (30 mg/kg) [33]. Thirty minutes before pilocarpine, scopolamine hydrobromide (1 mg/kg, i.p.) was injected to antagonize the peripheral cholinergic response caused by pilocarpine. Behavioral changes were observed, and seizures were evaluated by the Racine score [34]. Mice with seizures grade IV or V were used in subsequent experiments. After 60 min of status epilepticus (SE) in mice, we administered diazepam (10 mg/kg, i.p.) to stop seizures. Twenty-four hours after epilepsy, mice were deeply anesthetized with 2.5% sodium pentobarbital and decapitated. Brain tissues were quickly removed, and the bilateral hippocampus was isolated and stored at -80° C for subsequent assays of MDA, SOD, GSH-Px, and hippocampal DNA 8-OHdG. Part of the hippocampal tissue was and embedded in paraffin as described below for HE staining and *in situ* TUNEL staining.

### Epilepsy observation

According to the Racine epileptic mouse seizure criteria, seizure was classified as grade 0: no seizures; grade I: facial clonics, including rhythmic chewing, blinking, and whisker movement; grade II: convulsion mainly due to head nodding from movement of neck muscles; grade III: unilateral forelimbs with clonics and convulsions, but without erection of hindlimbs; grade IV; bilateral forelimbs with convulsions with erected body; grade V: generalized rigidity clonic seizures, stiffness in both hind limbs, body flexion, and falling. Successful modeling of epilepsy was defined as grade IV-V seizures after three consecutive pilocarpine challenges. The latency period was defined as the time between pilocarpine administration and the first IV-V grade episode. The time of episode was defined as extending from the onset of IV-V seizures to the end of the episode within 30 min after pilocarpine treatment. X of 90 mice died during status epilepticus.

### Hematoxylin and eosin (HE) staining

Mouse brain tissues were fixed in 10% formaldehyde for 24 h. Tissues were sectioned after embedding in paraffin. After dewaxing in xylene, tissues were dehydrated with gradient alcohol (100% I, 100% II, 95, 85, and 75%, for three min each). Hydrated tissues were immersed in hematoxylin staining solution for five min, and washed with water. Tissues were then immersed in 1% hydrochloric acid alcohol for 30 s, rinsed in running water for one min, immersed in 0.5% eosin dye for three min, rinsed in running water for one min, and dehydrated with gradient alcohol (75, 85, 95, and 100%, three min each). Tissues were cleared in xylene, sealed in neutral resin, and examined under a microscope.

### TUNEL staining

Apoptotic cells were stained by TUNEL after dewaxing and hydration of tissue as above. Tissues were then cut in 200 mL of 0.1 mol/L citrate buffer, pH 6.0, and heated to 90–95° C by 680 W (80% power) microwave for one min. Double-distilled water (80 mL, 20–25° C) was added for cooling. Sections were then washed with phosphate buffer solution (20–25° C) three times for five min each. Normal bovine serum (20%) was added for 30 min at room temperature. TUNEL reaction mixture (50 μL) was added to the sections and incubated at 37° C for 90 min (no TUNEL reaction mixture for negative controls). Tissues were washed by phosphoric acid buffer for three times for five 5 min. H_2_O_2_ methanol solution (3%) was then added at room temperature for ten min and then incubated at 37° C for 90 min, followed by addition of horseradish peroxidase (50 μL, POD) solution and incubation at 37° C for a further 30 min. Sections were washed with phosphate buffer three times for five min each time and then developed with diaminobenzidine/hydrogen peroxide (DAB/H_2_O_2_). Sections were then stained with hematoxylin, dehydrated as above, cleared, and sealed by neutral resin. Two slices from each animal were observed by microscopy, with counting of TUNEL positive (brown-stained) neurons in five randomly selected CA1 and CA3 regions.

### Immunohistochemistry

Paraffin brain tissue sections of mice were dried in a 60° C oven for 1 h. After drying, the sections were dewaxed with xylene for 10 min, dehydrated with 95%, 80% and 75% ethanol each for 1 min, washed with tap water for 1 min, incubated in 3% H_2_O_2_ (84885, Sigma, St. Louis, MO, USA) for 30 min at 37° C, and then boiled in 0.01 M citric acid buffer, at 95° C for 20 min, and cooled to room temperature. The sections were sealed with normal Goat serum working solution at 37° C for 10 min, incubated with primary rabbit anti HDAC5 (1: 250; ab1439), HIF1α (1:100; ab16066), and PFKB3 (1: 50; ab181861) at 4° C overnight. Next, the sections were incubated with Horseradish enzyme labeled Goat anti rabbit secondary antibody (DF7852, Shanghai Yaoyun Biotechnology Co., Ltd., Shanghai, China) at room temperature for 30 min. The sections were then developed by DAB, stained with hematoxylin, and sealed by neutral resin. Five high power visual fields (400×) were randomly selected from each section, and 100 cells were counted in each field to calculate the positive cell rate.

### Determination of MDA, SOD, GSH-Px concentrations

SOD, GSH-Px, and MDA concentrations were determined by commercially available test kits (Jiancheng, Nanjing, China). The thiobarbituric acid colorimetry method was used to detect MDA content. WST-1 method was used to detect SOD. GSH-Px was measured by UV colorimetry.

### Pro-inflammatory cytokines determined by ELISA

Contents of 8-OHdG (ab201734, Abcam, Cambridge, MA, USA) in hippocampal tissue or neuronal cells was determined by ELISA. The expression of IL-1β (ab197742, Abcam), TNF-α (ab208348, Abcam), and IL-6 (ab100712, Abcam) in hippocampal or neuronal cell culture supernatant was detected by ELISA [35].

### Hippocampal neuron epilepsy cell model

Mouse hippocampal neurons (Kamimi Yasuyo, Shanghai, China) were cultured in a neuron maintenance medium containing 98% neurobasal medium (Gibco, Waltham, MA, USA), 2% B27 supplement (Gibco), 0.2 M *L*-glutamine, 1 × 10^5^ U/L penicillin and streptomycin. Hippocampal neuronal maintenance medium was discarded and replaced with low-magnesium extracellular fluid (NaCl 145 mM, KCl 2.5 mM, HEPES buffer 10 mM, CaCl_2_ 2 mM, glucose 10 mM, and glycine 2 μM mmol/L dissolved in 1000 mL distilled water, filtered and sterilized, pH at 7.2, and stored at 4° C until use. Cells were washed and exposed to the low-magnesium extracellular fluid (2 mL/well, 37° C and 5% CO_2_) for establishing the hippocampal neuron epilepsy model [36]. After three h in this medium, the neurons were washed twice with maintenance medium, and then maintained in culture medium (2 mL). An EPC-10 patch clamp system (HEKA Electronik, Lambrecht, Germany) was used for voltage clamp recording in whole cells. As expected, preconditioning of hippocampal neurons without magnesium induced neurons to produce spontaneous repeated epilepsy-like discharges (SRED) that resemble those seen in human epilepsy [37].

### Cell transfection

Cells were transfected with the following plasmids (GenePharma, Shanghai, China): Blank (untreated mouse neuron cells), sh-NC, sh-HDAC5-1, sh-HDAC5-2, sh-HDAC5-3, epilepsy (hippocampal neuron epilepsy), epilepsy + sh-NC, epilepsy + sh-HDAC5, epilepsy + mimic-NC + oe-NC, epilepsy + miR-485 mimic + oe-NC, epilepsy + miR-485 mimic + oe-HDAC5, epilepsy + miR-485 mimic + oe-HIF1α, epilepsy + miR-485 mimic + oe-PFKFB3, epilepsy + sh-NC + oe-NC, epilepsy + sh-HDAC5 + oe-NC, epilepsy + sh-HDAC5 + oe-HIF1α, epilepsy + sh-HDAC5 + oe-PFKFB3.

Lentiviral supernatant was prepared by mixing 1.5 μg packaging mixed plasmid, 0.5 μg expression plasmid, and 250 μL serum-free medium in a 1.5 mL sterilized tube at room temperature for five min. Lipofectamine 2000 was dissolved in 250 μL serum-free medium in a 1.5 mL sterilized EP tube and incubated at room temperature for five min. DNA solution and liposome solution were gently mixed. 293T cells were digested with trypsin for 20 min at room temperature. Cells were resuspended in serum-containing medium. In each well of a six-well plate, 1 mL of serum-containing growth medium was added, followed by DNA-liposome complex. Resuspended 293T cells (1 mL, 1 × 10^6^ cells/mL) was added to the wells. Cells were incubated in a CO_2_ incubator at 37° C overnight and the medium replaced. Supernatant was harvested 48–72 h after transfection and centrifuged at 3,000 rpm for 20 min. Twenty-four hours before transfection, mouse hippocampal neurons (at 80–90% confluence) were seeded in 6-well culture plates and transfected with miR-485 mimic or control (Lipofectamine 2000, Sigma).

### RT-qPCR

Total RNA was extracted by Trizol (15596026, Invitrogen, Carlsbad, CA, USA). RNA concentration and purity were measured by Nano-Drop ND-1000 spectrophotometer. RNA was reverse-transcribed to cDNA by PrimeScript RT reagent kit (RR047A, Takara, Kusatsu, Japan) and TaqMan^®^ MicroRNA Reverse Transcription Kit (Applied Biosystems, Foster City, CA, USA). Primer sequence are presented in Table 3 (Sangon, Shanghai, China). cDNA was subjected to real-time PCR (SYBR® Premix Ex TaqTM II, TaKaRa, Dalian, China) by a fluorescent quantitative PCR system (ABI 7500, Foster City, CA, USA). GAPDH was used as an internal reference. Reaction conditions were as follows: pre-denaturation at 95° C for 30s, denaturation at 95° C for 30 s, annealing at 20° C, extension at 72° C for 30 s, for 40 cycles. A miR-485 kit (assay ID: 001036, Applied Biosystems) was used to detect miR-485 expression. U6 (assay ID: 001973, Applied Biosystems) was used as an internal reference, and relative expression of target genes was calculated by the 2^-ΔΔCt^ method [38].

**Table 3:** Primer sequences for RT-qPCR.

**Targeted gene**	**Forward primer (5'-3')**	**Reverse primer (5'-3')**
HDAC5	AGCACCGAGGTAAAGCTGAG	GAACTCTGGTCCAAAGAAGCG
miR-485	AGAGGCTGGCCGTGATG	GTGCAGGGTCCGAGGT
U6	GCTTCGGCAGCACATATACTAAAAT	CGCTTCACGAATTTGCGTGTCAT
GADPH	GGAGCGAGATCCCTCCAAAAT	GGCTGTTGTCATACTTCTCATGG

### Western blotting

Total protein from hippocampal neuronal cells was extracted using RIPA lysis buffer (Sigma). Nuclear protein lysates were prepared by Qproteome Cell Compartment Kit (QIAGEN, Hilden, Germany). The Protein concentration was determined by BCA kit (Thermo Scientific, Waltham, MA, USA). Proteins (20 μg) were separated by 10% SDS-PAGE and transferred to a PVDF membrane (Millipore, Burlington, MA, USA). Membranes were blocked with 5% skim milk at room temperature for one h. Membranes were washed with PBS and then incubated with primary antibodies against HDAC5 (1:1000, ab1439, Abcam), HIF1α (1:1000, ab179483, Abcam), PFKFB3 (1:1000, ab181861, Abcam) at 4° C overnight. Membranes were washed in PBS at room temperature (three times for five min each). Membranes were then incubated with horseradish peroxidase (HRP)-labeled goat anti-rabbit IgG antibody (1:200, ab97051, Abcam) for 1 h at 37° C with shaking. Membranes were washed again three times in PBS, and then immersed in an ECL reaction solution at room temperature for one min. The protein bands were developed in the dark, and the gray scale intensity for each protein was analyzed by Image J. The experiment was repeated three times independently.

### Dual luciferase reporter gene assay

Target gene analysis of HDAC5 and miR-485 was performed by an online website. Dual luciferase reporter gene assay was used to verify the targeting relationship between miR-485 and HDAC5. The dual luciferase reporter gene vector of HDAC5 and with mutation of the predicting binding site to miR-485 were constructed: pGL3-HDAC5 Wt and pGL3-HDAC5 Mut. Two reporter plasmids were co-transfected with miR-485 mimic, mimic-NC, and pRL-TK (internal reference plasmids expressing Renilla luciferase) in HEK293 cells for 24 h by TransDetect Double-Luciferase Reporter Assay Kit (FR201-01, Transgen Biotech, Beijing, China). Cells were lysed and supernatant collected. Luciferase activity was determined by Dual-Luciferase Reporter Assay System (E1910, Promega, Madison, WI, USA). Luciferase Reaction Reagent (100 μL) was equilibrated at room temperature to the test tube and mixed with 20 μL of cell lysate. The activity of firefly luciferase was measured. Luciferase Reaction Reagent II was used for Renilla luciferase. Relative luciferase activity between firefly and Renilla luciferase was calculated.

### Immunofluorescence

Neurons were seeded on poly-*L*-lysine-treated slides. When cell density reached 50% confluence, cells were washed three times with PBS. Cells were then fixed with 4% paraformaldehyde for 30 min at room temperature. After washing three times with PBS and permeation with 2% Triton X-100 for 15 min, cells were further permeabilized with 2 M HCl for 20 min and washed three times with PBS. Cells were blocked with 2% BSA for 45 min and then incubated with HIF1α antibody (1:200, ab216842, Sigma) at 4° C overnight. After PBS washing, cells were incubated with goat anti-rabbit IgG H and L fluorescent secondary antibody (1:1000, ab6717, Abcam) at room temperature for 2 h, followed by three washes in PBS. After staining with DAPI (2 μg/mL), expression of NF-200 was observed under an upright fluorescence microscope.

### Immunoprecipitation (IP)

Cells were collected and incubated on ice for 30 min with cell lysate (Beyotime Biotechnology, Shanghai, China) and centrifuged at 4° C for 10 min to obtain the supernatant. One part of the lysed cells was added with HIF1α antibody (ab16066, 1: 1000, Abcam) and incubated overnight at 4° C. 10 μL protein A agarose beads was washed with lysis buffer for 3 times by centrifugation at 3000 rpm for 3 min each time. The pretreated beads were added into the cell lysate incubated with antibody overnight, and then slowly shaken at 4° C for 2–4 h to couple the antibody with the beads. After IP reaction, the beads were centrifuged at 4° C at a speed of 3000 rpm for 3 min to the bottom. The supernatant was carefully aspirated, and beads were washed 3–4 times with 1 mL lysis buffer. Finally, 15 μL of 2 × SDS loading buffer was added and boiled for 5 min, which was used for subsequent Western blot detection [39].

### Chromatin immunoprecipitation (ChIP)

CHIP kit (Millipore) was used. After reached 70–80% confluence, 1% formaldehyde was added to the cells and fixed for 10 min at room temperature to crosslink the DNA and protein. After crosslinking, the samples were randomly fractured by ultrasonic treatment for 120 w, 2 s on, 5 s off for 15 times to break into segments of appropriate size. The supernatant was obtained by centrifugation at 13000 rpm at 4° C and then divided into three tubes. The three tubes were added with positive control antibody RNA polymerase II, NC antibody normal human IgG, and rabbit anti HIF1α (1: 100; ab2185, Abcam) respectively, and incubated overnight at 4° C. Protein agarose/sepharose was used to precipitate the endogenous DNA protein complex. After centrifugation, the supernatant was removed, the nonspecific complex was washed, and crosslinking was performed overnight at 65° C. The DNA fragment was extracted and purified by phenol/chloroform. The expression of PFKFB3 promoter was detected by RT-qPCR. Each experiment was repeated three times [40].

### Flow cytometry

Based on Annexin V Apoptosis Detection Kit (Biosea, Beijing, China), neuronal cells were treated with 0.01, 0.10 and 1.00 nM LDM for 48 h. Cells were collected by centrifugation at 120 × g for ten min at 4° C, and washed with PBS 2–3 times. Cells were resuspended in 150 μL binding buffer, which was added V-FITC (10 μL) and PI (5 μL) with gentle mixing at room temperature in dark. After 15 minutes of reaction, 150 μL of binding buffer was added. Cell apoptosis was analyzed with flow cytometer (Coulter Electronics, Brea, CA, USA). Fluorescence intensity was recorded and analyzed by FACScan software.

### Statistical analysis

Data were analyzed by SPSS 21.0 (IBM, Armonk, NY, USA). Data are expressed as mean ± standard deviation. Data between two groups were compared independent sample t-test. Data between multiple groups were compared by one-way analysis of variance and post-hoc Tukey’s test. Differences were considered significant when *p* < 0.05.

## Supplementary Material

Supplementary Figures
