# Supplementary material for: miR-485 inhibits histone deacetylase HDAC5, HIF1α and PFKFB3 expression to alleviate epilepsy in cellular and rodent models
Source: Aging (Albany NY). 2021 May 21;13(10):14416–32. doi: 10.18632/aging.203058 (PMC8202868; doi:10.18632/aging.203058)
Supplement: Supplementary Figures [file aging-13-203058-s001.pdf]

SUPPLEMENTARY FIGURES

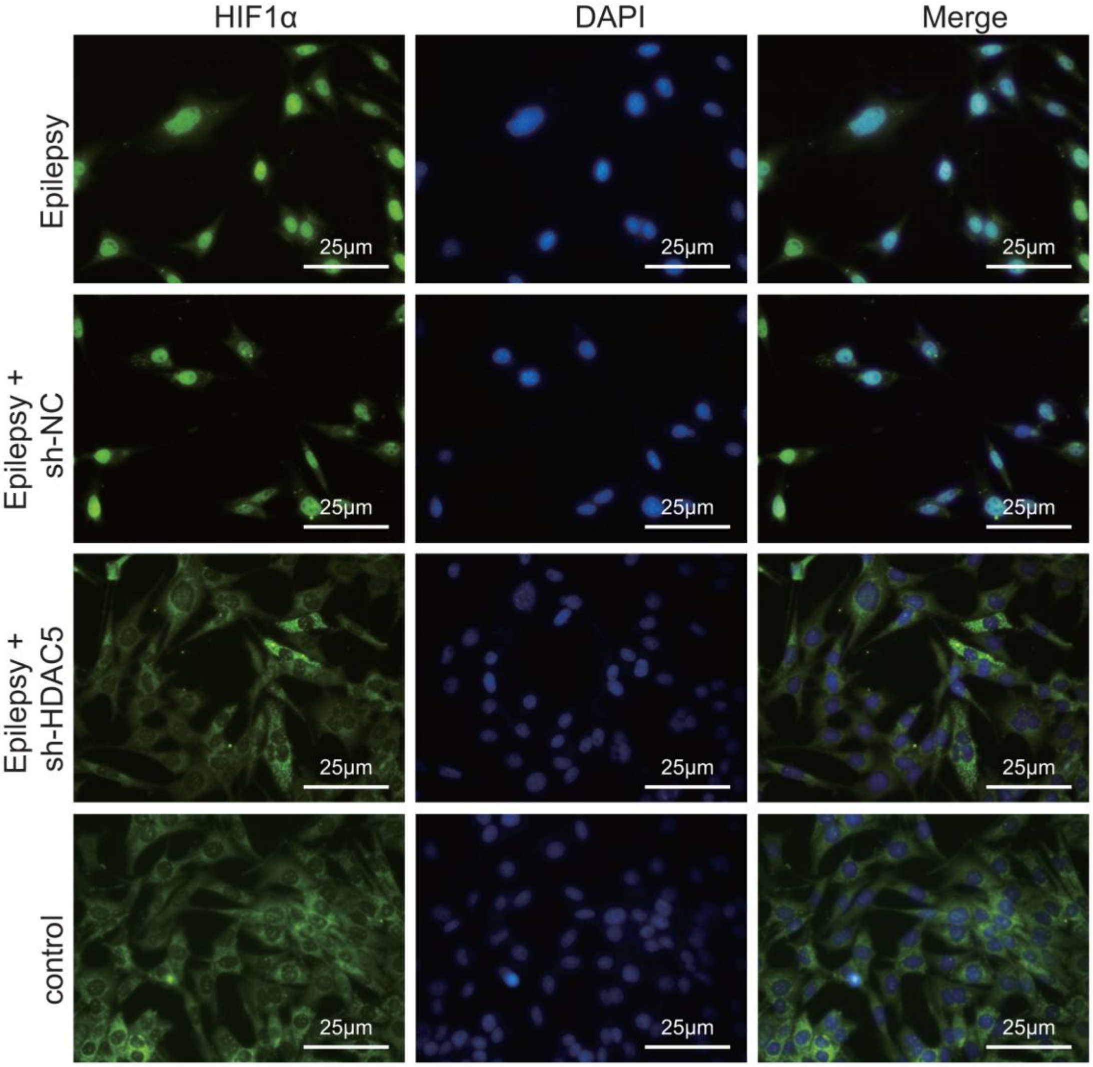

Supplementary Figure 1. Representative immunofluorescence micrographs showing the expression of HIF1α (400 ×).

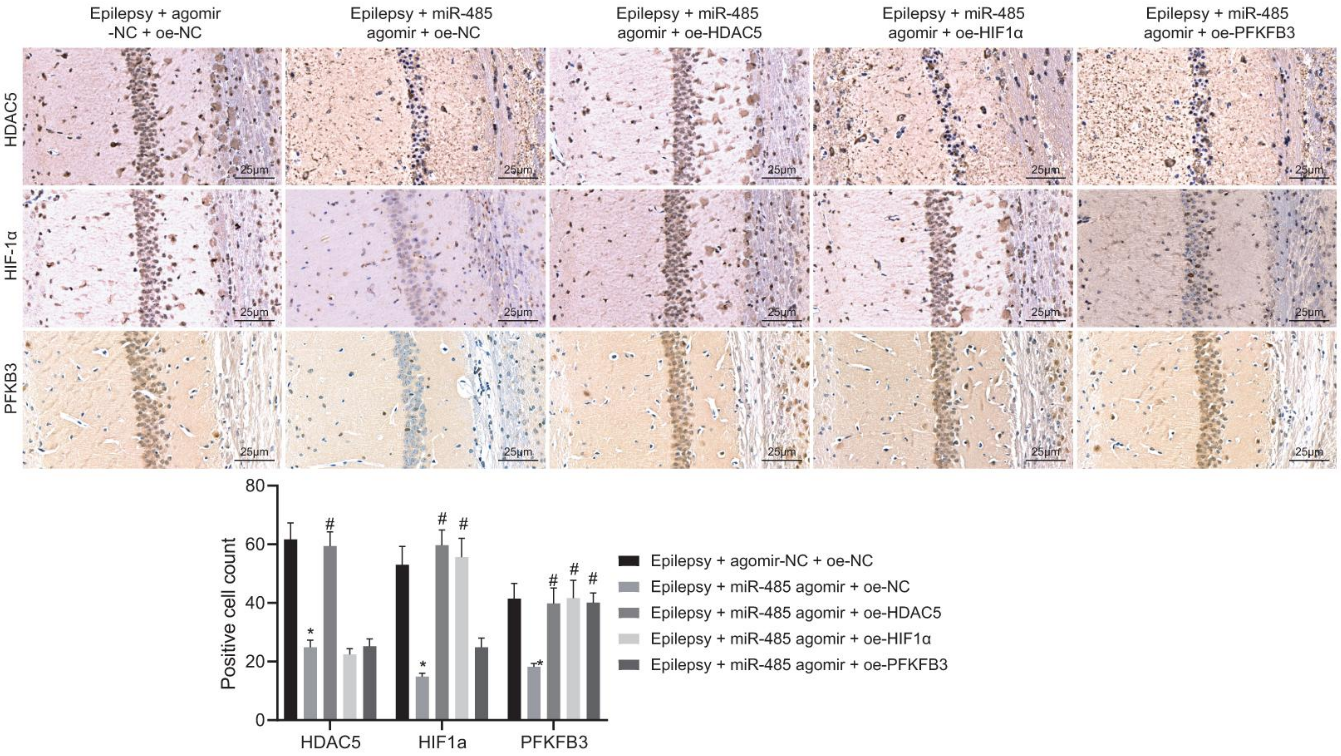

Supplementary Figure 2. The expression of HDAC5, HIF1α, and PFKFB3 in hippocampus detected by immunohistochemistry.
